# How Vaccination Rumours Spread Online: Tracing the Dissemination of Information Regarding Adverse Events of COVID-19 Vaccines

**DOI:** 10.3389/ijph.2022.1604228

**Published:** 2022-05-30

**Authors:** Tauel Harper, Katie Attwell

**Affiliations:** School of Social Sciences, University of Western Australia, Perth, WA, Australia

**Keywords:** social media, COVID-19, media, information dissemination, adverse events, infodemic, communication, rumor control

## Abstract

**Objectives:** To trace the emergence and dissemination of the most prominent rumours about potential adverse effects of COVID-19 vaccines.

**Methods:** We use a weekly Google Trends search to gather information about what alleged adverse events are being associated with COVID vaccines by the general population. We then use CrowdTangle and Factiva searches to examine how discussions about the five most prominent adverse events have spread through traditional media channels and Facebook.

**Results:** Traditional mass media reporting remains crucial in both promoting and moderating discussions around alleged adverse events. While some cases illustrate that social media networks can synthesise and amplify rumours about adverse events, traditional media coverage remains crucial as a forum for exploring and debunking spurious claims.

**Conclusion:** Traditional media stories still bear signficant responsibility as credibility markers for rumours about vaccine adverse events. Journalists should therefore be encouraged to be particularly earnest when reporting such stories, and the scientific community should aid journalists in this task by clearly responding to any rumours emerging online.

## Introduction

With the advent of COVID-19, health authorities have diagnosed an “infodemic” characterised by misinformation and disinformation circulating social media [1]. With the roll-out of vaccination programs, adverse events following vaccination have occurred. However, many more unfounded rumours have also disseminated. This study establishes how rumours of alleged adverse events have been disseminated around the world during the first months of the roll-out. A growing body of literature identifies information about alleged adverse events as critical drivers of vaccine hesitancy [2, 3], with a 2019 study suggesting “introducing a small risk of a vaccine adverse event may significantly prolong the tail of an outbreak” [4]. There is particular concern that social media may proliferate misinformation about adverse events and therefore stimulate vaccine hesitancy [5].

We used social network analysis and digital trace data to examine the origin and spread of rumours about adverse events following COVID-19 vaccinations, presented as narrative case studies. This study sits within “Coronavax: Preparing Community and Government,” which seeks to uncover the conditions for a successful vaccination program in Western Australia [6].

## Methods

Our mixed methods approach mobilised two distinct stages of data gathering. First, we established emergent concerns about alleged adverse events associated with COVID-19 vaccinations by using Google Trends. Google Trends uses the metric “Relative Search Volume” (RSV) to track changes in the volume of searches for particular topics or search terms on Google. Its ability to discern changes in search activity makes Google Trends a useful tool for identifying the outbreak of public rumours [7–9].

Our investigation extended from 1 December 2020 (to cover the beginning of the vaccination program in the United Kingdom and United States) until 21 April 2021. During this time we searched Google Trends data worldwide weekly, interrogating the search topic “COVID-19 vaccine—pharmaceutical” and examining related queries and topics for increased mentions of possible adverse events. This process revealed twelve alleged adverse events associated with searches on the COVID-19 vaccine during the period. Of these, ten were easily identifiable: Bell’s palsy, sterilisation, cerebral palsy, facial paralysis, syncope, coma, hematologic disease, asthma, thrombus, and coagulation. Two further topics alluded to possible adverse events: the topic “mammography” registered on 18 February 2021, seemingly related to lymph node swelling; finally, the topic “Hank Aaron” was associated with the vaccine due to this famous American baseball player dying 2 weeks after receiving his vaccination. We included “Hank Aaron,” as this unique identifier allowed us to trace rumours of death as an adverse event.

**FIGURE 1:**
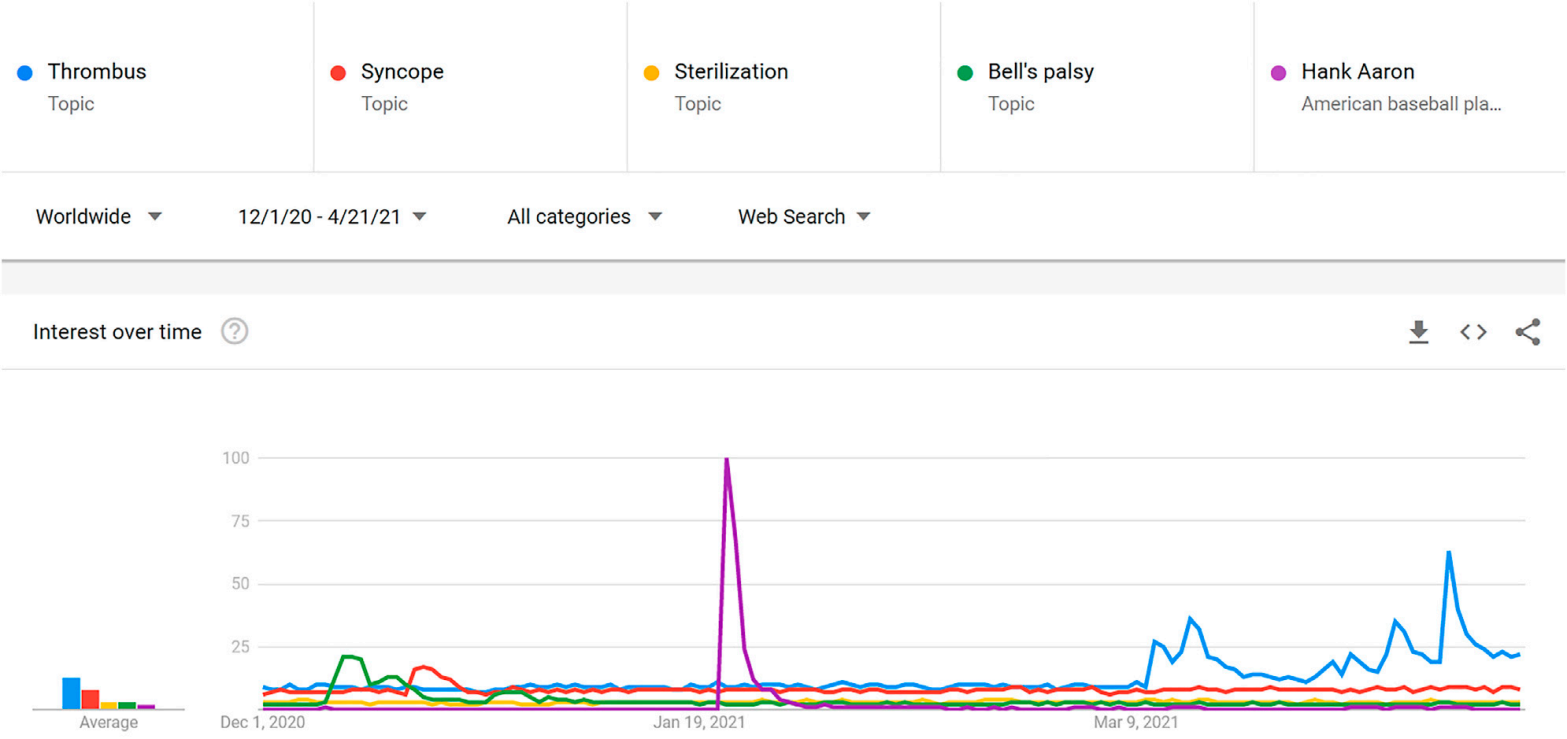
Google Trends Comparison Showing Relative Search Volumes for Thrombus, Syncope, Sterilisation, Bell’s palsy and Hank Aaron from 1 December 2020 to A21 April 2021 (How vaccination rumours spread online, worldwide, 2020–2021).

We then compared the Google Trends data, selecting topics showing significant spikes in search volume during the study period. The resulting set of four topics included thrombus (clotting), syncope (fainting), coagulation (clotting), Bell’s palsy, and Hank Aaron (death). Turning to the remainder of cases, sterilisation had the highest search volume, so we included it as a study of persistent rumours. Search patterns for “thrombus” and “coagulation” clearly mirrored each other as references to blood clotting issues, but Google Trends’ language processing parsed various searches about clotting into either topic. As these trends were associated by epidemiologists with the emergence of “clotting” as a rumoured adverse event, we collapsed them. This left a final list of five rumours to investigate: clotting (thrombus and coagulation), fainting (syncope), sterilisation, Bell’s palsy, and death (Hank Aaron) (see Figure 1).

Our second stage involved examining both social and traditional media to discover stories or posts that may have generated these rumours. We used CrowdTangle, which is freely available for academic research, enabling researchers to interrogate public facing data from all public pages, groups, and profiles on Facebook. CrowdTangle cannot be used to examine how news spreads on personal profiles and only tracks public engagements rather than views or imprints; nevertheless, it provides a reasonable overview of the dissemination of general trends and large stories. Facebook was chosen because it is the most widely used social media platform and the most commonly used for news sharing [10, 11]. CrowdTangle also allowed for Boolean searching within defined dates and without interference from personal or geographic information, which may otherwise have prejudiced our results. We conducted global searches of CrowdTangle using the terms and dates derived from stage one. Search terms were limited to English and based upon “common” usages (“fainting” instead of syncope; “clotting” instead of thrombus and coagulation). Our use of these datasets is approved by the Child and Adolescent Health Service Human Research Ethics Committee permit number RGS0000004457.

We then searched for the same terms and date ranges on Factiva, a database facilitating targeted searches of news reports from >200 countries. This allowed us to cross-check whether stories were originating on traditional or social media and understand which spread online (or did not). Once again, Factiva allowed for Boolean searching within the English language corpus.

By searching CrowdTangle and Factiva using “Covid vaccine” and “(rumoured adverse effect OR common parlance alternative)” around the Google Trends spike dates, we identified how particular stories broke chronologically and were then distributed around social and traditional media. Searching day by day before a spike isolated the first public post and news story to mention both the alleged adverse event and the vaccine. We conducted CrowdTangle searches for the same terms over the duration of the Google Trends “spike,” generating data files that listed posts chronologically (earliest-first) to organise sequence. We then searched along the same parameters but ordered by most interactions (likes, comments, and shares) to measure impact. More targeted searches were then used to find out more about specific sources within CrowdTangle, which could show numbers of shares and channels of distribution for specific posts. We then developed case studies on how the five rumours about clotting, fainting, sterilisation, Bell’s palsy and death spread around social media networks.

## Results

### Clotting (Thrombus and Coagulation)

**FIGURE 2:**
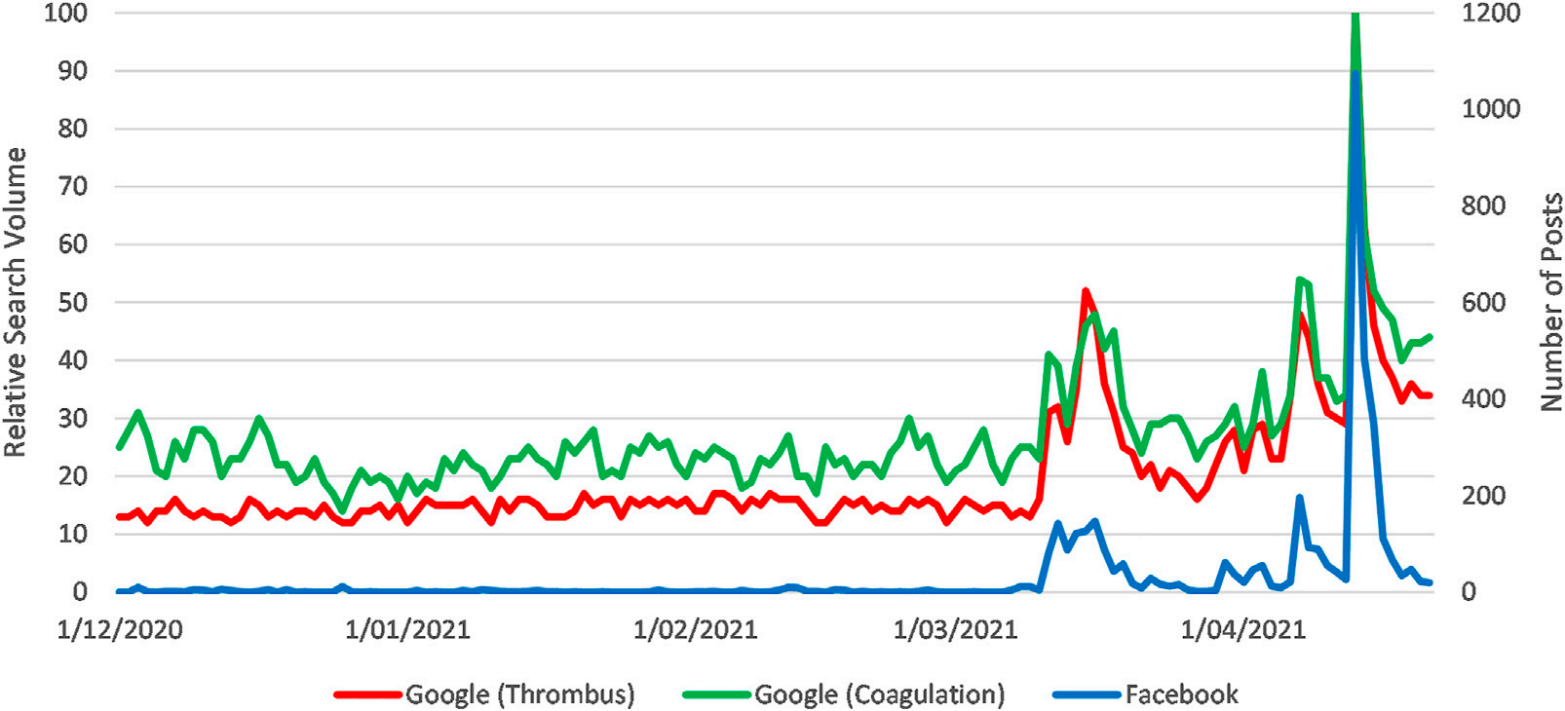
Sparklines for Google RSV for the search topics “Thrombus” and “Coagulation” and Number of Facebook Posts with the Search Terms “Covid Vaccine AND (Clotting OR Thrombus OR Coagulation OR Clot)” from 1 March to 21 April 2021 (How vaccination rumours spread online, worldwide, 2020–2021).

Clotting was discussed in 4,153 posts which posts received 300,069 interactions (likes, shares, comments etc.) at an average rate of 72 interactions per post. There was a strong correlation between Google RSV for thrombus and coagulation and number of public Facebook posts on clotting (Pearson’s r = 0.86) (see Figure 2) [12].

The initial increase in Google Trends RSV after 7 March can be attributed to traditional media reports that Austria was suspending its AstraZeneca (AZ) roll-out after a nurse developed “clotting/embolism” post-vaccination and subsequently died. This news was initially reported by Reuters and cited by the APA and Austrian broadcaster ORF.

The initial growth in Facebook interactions took place after the Reuter’s report was shared widely—with the first posts (citing the Reuters story) appearing on Facebook pages in Uganda, India, and the Philippines on 7 March. The Reuters story received significant media attention and was published in traditional media outlets in Russia, United Kingdom, Latin America, China, and Canada on the same day. Posts citing reports from Sky News and Russia Today were the most widely shared, reaching locations as dispersed as Ghana, Lebanon, the United States, and New Zealand before the story was covered by (English speaking) traditional media in those countries. While these media reports generated 779 Facebook interactions on 8 March, by 9 and 10 March social media engagement with the Reuter’s story had dropped close to zero. However, nine interactions from four posts on 10 March grew to 9,867 interactions with 142 posts by 12 March, as traditional media reported that more EU countries had started suspending the AZ roll-out.

Online interaction with this topic remained constant, never dropping lower than the 706 interactions on 27 March. There was an increase in activity of similar magnitude to the EU announcements around 6 April when an Italian newspaper reported “a Senior official” at European Medicines Agency (EMA) asserting a link between the AZ vaccine “and extremely rare cases of blood clots” [13]. The mass media covered this story extensively, and it was also distributed widely by both pro- and antivax groups on Facebook (vaccine sentiment was identified by pages and groups’ titles). The spike in Google Trends on 13 April was triggered when the US Department of Health recommended pausing the Johnson & Johnson vaccine while rare blood clotting cases were reviewed. This was extensively covered by US mainstream media, notably the Wall Street Journal and MSNBC, whose stories received the most social media interactions on this topic.

### Fainting (Syncope)

**FIGURE 3:**
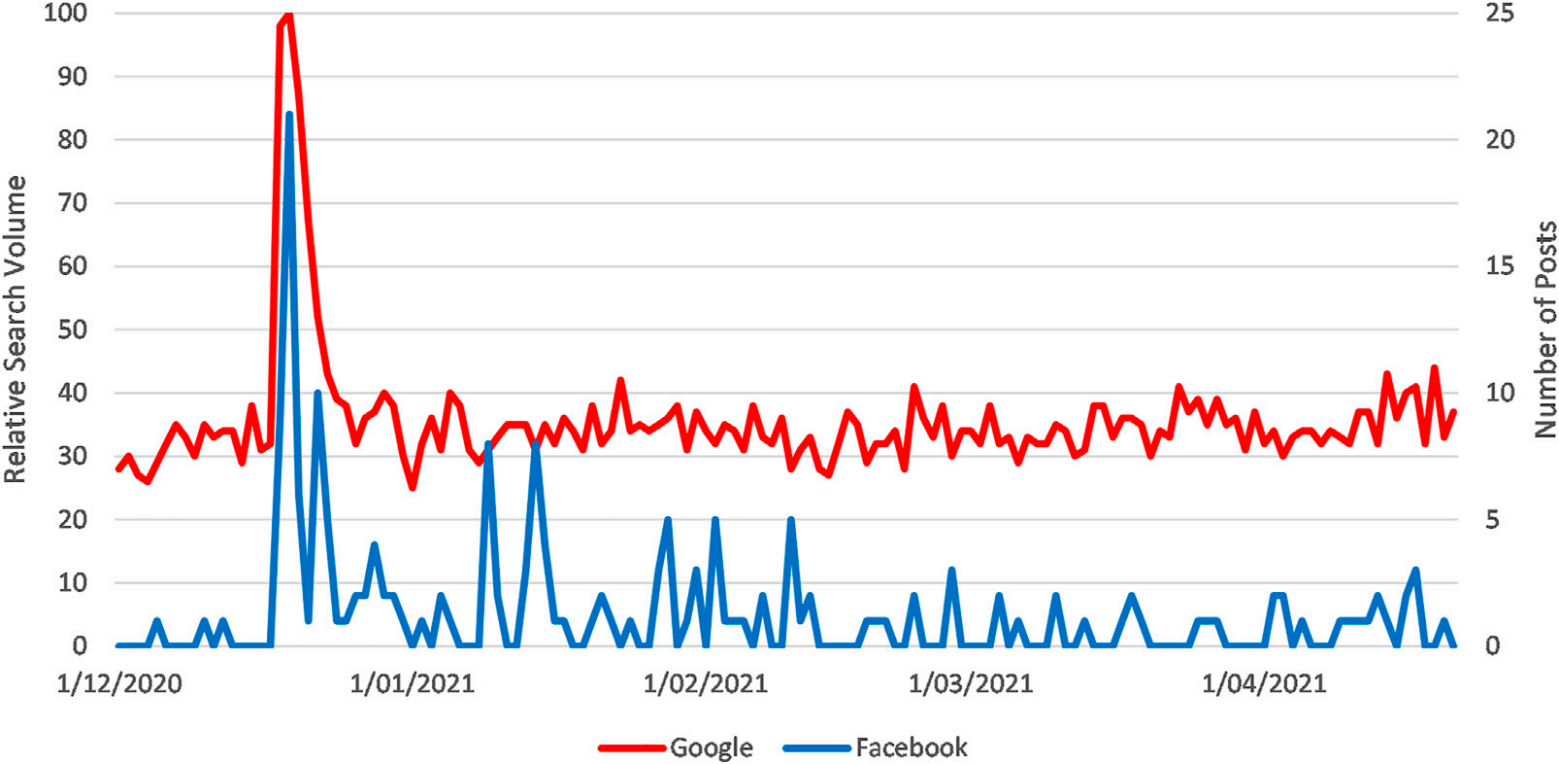
Sparklines for Google RSV for the search topic “Syncope” and Number of Facebook Posts with the Search Terms “Covid Vaccine AND (Fainting OR Syncope)” from 1 March to 21 April 2021 (How vaccination rumours spread online, worldwide, 2020–2021).

Fainting was discussed in 175 posts, receiving 20,780 interactions at an average rate of 118 interactions per post. There was a strong correlation between Google RSV for syncope and number of public Facebook posts on the covid vaccine and fainting (Pearson’s r = 0.65) (see Figure 3) [14].

On 17 December a nurse fainted live on a local news streaming video in Chattanooga, Tennessee (WRCB Channel 3) shortly after receiving the Pfizer vaccine. This story was reported on traditional and social media; social media activity and Google searches dramatically increased shortly afterward. Traditional media reports in the United States, United Kingdom, Australia, China and Latin America included the nurse’s own disclosure that she had a history of fainting and cautioned against attributing the fainting episode to the vaccine. The most shared Facebook post about this rumour was from the local news organisation that originally aired the event and included an explanation from the nurse about this pre-existing condition. Despite this, many social media posts either failed to mention her pre-existing condition or derided her “disclosure” as a cover up; the second most shared post came from a public figure who cited the story incredulously. The story was shared over the next couple of days, and vaccine opponents continued to frame it as evidence of an adverse event for many weeks afterward. A small increase in social media activity on 26 December resulted from further unsubstantiated rumours, spread only on social media, that the nurse had died. Once again, while several traditional media organisations covered this issue, they only did so to state that the death rumours had been disproved.

### Infertility (Sterilisation)

**FIGURE 4:**
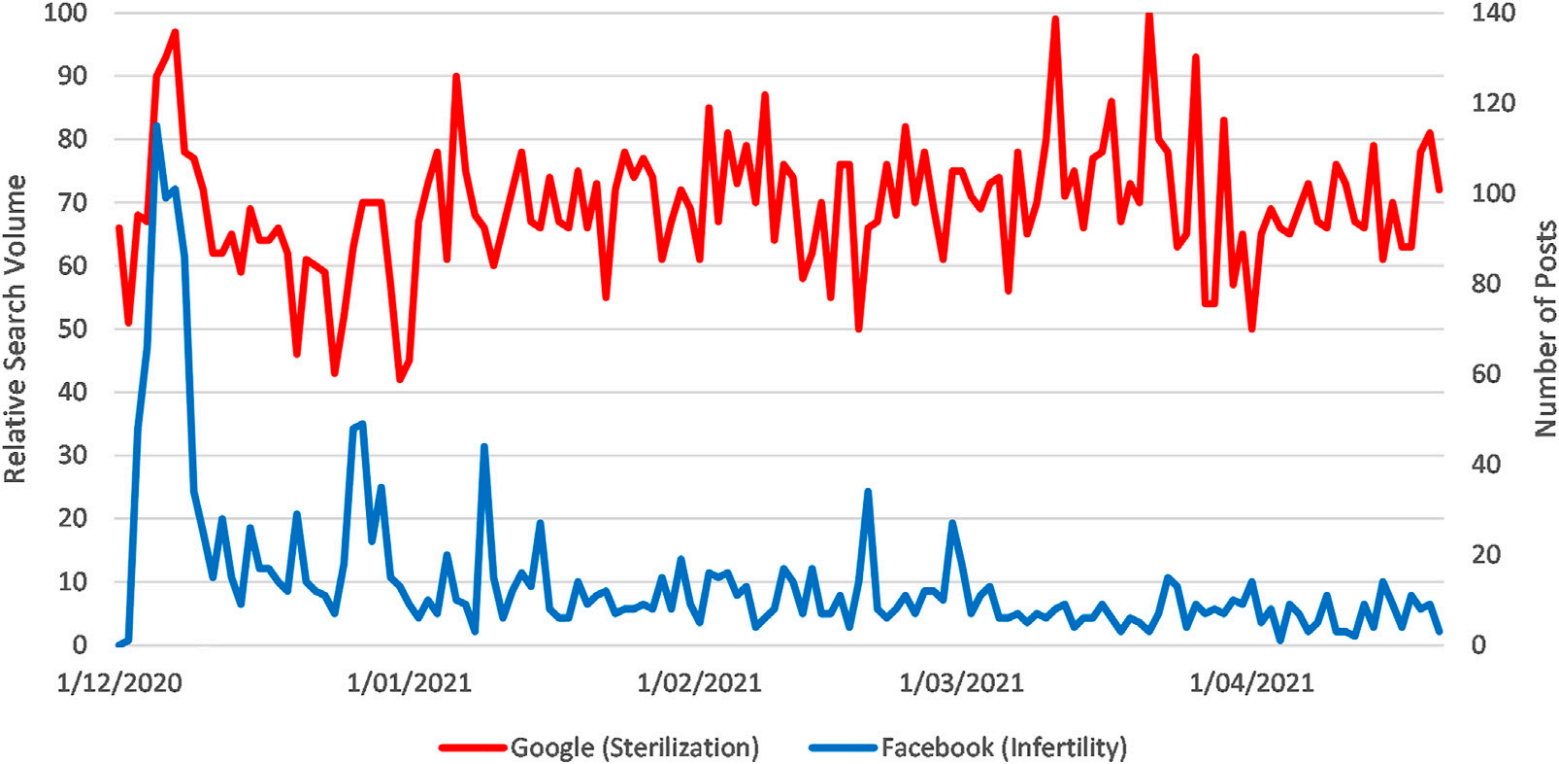
Sparklines for Google RSV for the search topic “Sterilization” [fertility] and Number of Facebook Posts with the Search Terms “Covid Vaccine AND (Sterilization OR Sterilisation OR Fertility OR Infertility)” from 1 March to 21 April 2021 (How vaccination rumours spread online, worldwide, 2020–2021).

Sterilisation was discussed in 2,075 posts which received 143,655 interactions at an average rate of 69 interactions per post. There was a small correlation between Google RSV for sterilisation (as a topic related to fertility) and the number of public Facebook posts on the covid vaccine and sterilisation or infertility (Pearson’s r = 0.22) (see Figure 4) [15].

Google searches about sterilisation (loss of fertility) increased significantly after a flurry of social media posts in early December 2020. After that initial increase, search activity has remained constantly high. “Covid vaccine sterilization” is the highest search query related to the (fertility associated) topic “sterilization” during our period of observation. It was also among the first alleged adverse events to be associated with the vaccine.

The initial growth in social media attention for “sterilization” in December 2020 was the result of two stories relating to sterilisation and infertility being shared widely on social media. The first featured a video of a vaccine scientist stating in a UK television interview (recorded in August 2020) that any vaccine was “unlikely to sterilize an entire population” but would only be effective in 60%–70% of the population [16]. The scientist later clarified that his effectiveness claim was “referring to the ability of the vaccines to completely eliminate viral replication…[and not] to fertility” [17]. A YouTube video of this statement was created on 29 November 2020, but it was not initially shared widely. However on 3 December a second story emerged from a personal health and wellness blog with the confected headline “Head of Pfizer Research: Covid Vaccine is Female Sterilization”. The story misrepresented a letter of concern written by two vaccine experts, one of whom had worked at Pfizer 9 years earlier, to the European Medicines Agency about the possibility of fertility complications [18]. The blog post spread rapidly through vaccine-sceptical communities on social media, who subsequently shared the video of the vaccine scientist speaking of “sterilization” as well.

Generally, traditional media only covered this story to point out that these two social media stories misrepresented the truth. Similarly, many of the most shared and interacted posts on social media were from fact checkers pointing out false claims. Two traditional media reports connected covid vaccine hesitancy and historical attempts to sterilize Black and Indigenous communities; these were not widely shared but a post on this topic from a public individual has subsequently received more than 4,000 interactions [19, 20]. From 17 February, traditional media sources also covered the story of a New York City waitress who refused the vaccine because of concerns about its effect of fertility. While this led to more social media interactions on this topic, there was no corresponding increase in Google search volumes.

There was an increase in interactions on this topic on 19 January due to a meme spreading (particularly in South Africa) about “abortion drugs being found in Bill Gates’ tetanus vaccine,” with antivax communities posting this as evidence that the COVID vaccine could not be trusted. Once again, traditional journalists generally only discussed this rumour to debunk it. There was another increase in interactions on 12 April after The Daily Mail (UK) reported that vaccine recipients were experiencing changes in their menstrual cycles [21], a story spread widely by vaccine-hesitant networks.

### Bell’s Palsy

**FIGURE 5:**
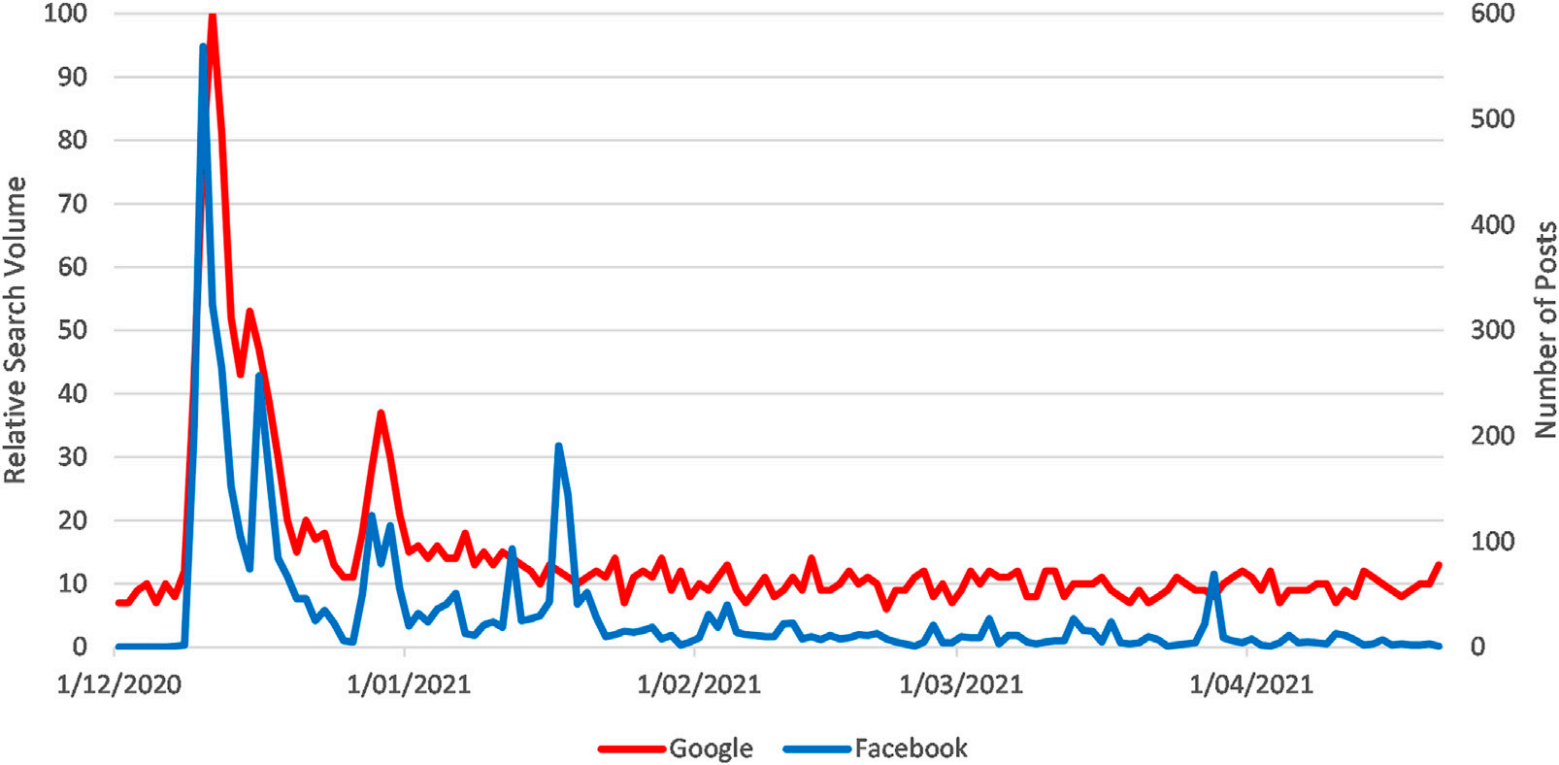
Sparklines for Google RSV for the search topic “Bell’s palsy” and Number of Facebook Posts with the Search Terms “Covid Vaccine AND (Bell’s palsy OR Bells palsy OR Facial Paralysis)” from 1 March to 21 April 2021 (How vaccination rumours spread online, worldwide, 2020–2021).

Bell’s palsy was discussed in 4,735 posts receiving 255,789 interactions at an average rate of 54 interactions per post. There was a strong correlation between Google RSV for Bell’s palsy and the number of public Facebook posts on the covid vaccine and Bell’s palsy (Pearson’s r = 0.86) (see Figure 5) [22].

On 8 December the US Food and Drug Administration released a briefing document outlining results for 38000 trial participants for the Pfizer vaccine, identifying “no specific safety concerns”. Nevertheless, news blogs from Bangladesh and the US focused on the detail that four participants developed Bell’s palsy. This was then reported 3 h later by The Daily Mail (UK), with the headline “Four volunteers who got the Pfizer vaccine developed Bell’s palsy—but the FDA denies that the temporary facial paralysis was caused by the shot” [23]. That one Daily Mail story was the only traditional news story to cover the FDA results negatively, yet it was shared copiously around the world—the 13 top posts on the topic that day all cited the online story. Most were shared by pages or profiles in the US but also by pages based in France, Israel, and India. Resultingly, the number of interactions on Facebook posts about Bell’s palsy went from 18 globally on the 8th to 24083 on the 9th.

The second hump around 15 December seems to have been driven by traditional media sources that had waited for the publication of the FDA report). Again, traditional media coverage tended to dismiss Bell’s palsy as a serious concern [24]. On 28 December a prominent public doctor debunked concerns about Bell’s palsy in two videos that received more than 3,300 interactions, which led to an increase in post numbers and RSV. Interestingly, a similarly widely shared post from RT News about the number of cases of Bell’s palsy in Israel generated even more interactions on social media but did not lead to a corresponding increase in RSV.

### Death (Hank Aaron)

**FIGURE 6:**
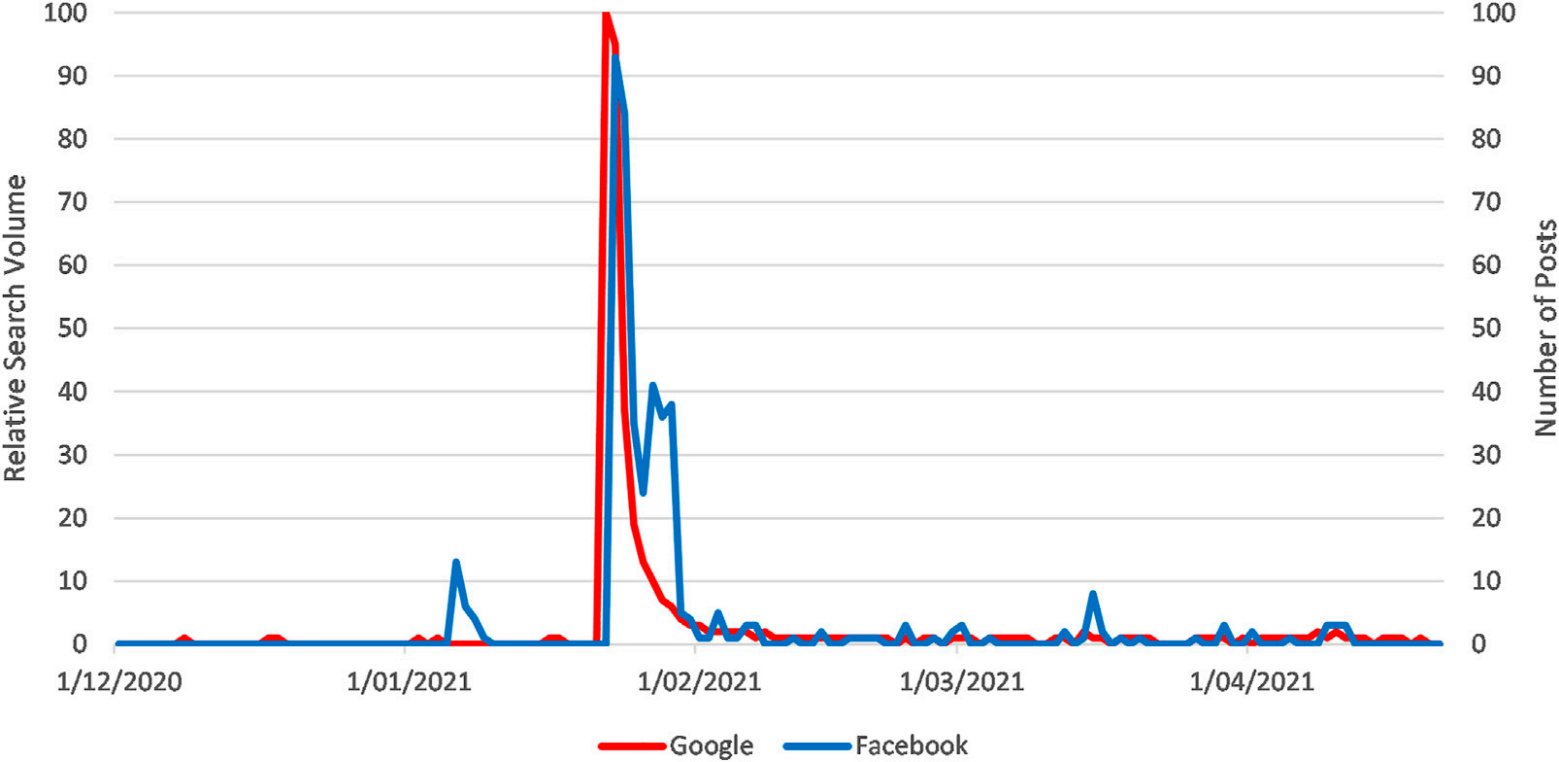
Sparklines for Google RSV for the search topic “Hank Aaron” and Number of Facebook Posts with the Search Terms “Covid Vaccine AND Hank Aaron” from 1 March to 21 April 2021 (How vaccination rumours spread online, worldwide, 2020–2021).

Hank Aaron was discussed in relation to the vaccine in 447 posts receiving 36,560 interactions at an average rate of 82 interactions per post. There was a strong correlation between Google RSV for Hank Aaron and the number of public Facebook posts on the covid vaccine and Hank Aaron (Pearson’s r = 0.61) (see Figure 6) [25].

On 5 January 2021, 86-year-old Hank Aaron got his covid vaccine on camera in the hope of inspiring other Black Americans. This story was reported by the Associated Press on 6 January, and TMZ.com coverage of the event was shared 280 times. Aaron’s death on 22 January was attributed to natural causes [25], but was quickly associated with his vaccination on social media and by some online news outlets [26].

While traditional media covered his death without attributing it to the vaccine, one online news story explicitly connecting them was shared 12 times. That story was subsequently cited in a second blog post [27] that proliferated throughout social media—being shared 12,472 times. A single repost by US anti-vax activist Robert F Kennedy Jr was shared more than 4,200 times alone. On 23 January a meme attributing Aaron’s death to the vaccine spread widely; further memes derived from the CBS headline from 6 January “Hank Aaron, 86, receives COVID-19 vaccine and hopes to inspire other Black Americans to do the same” with various sarcastic notes added.

The spread of rumours prompted traditional media sources such as MSNBC, TMZ and the Atlanta Journal-Constitution to deny any link between Aaron’s death and his vaccination; particularly after the Atlanta Journal-Constitution published the medical examiner’s statement that he had died of natural causes. While these stories were shared widely, Robert F Kennedy Jr’s post was shared 4.7 times more on social media than the next most shared post by MSNBC (893 shares). The “traditional” media story most shared among anti-vax groups (1222 times) was an Indian news report from Republicworld.com that led with the slightly hyperbolic headline “Hank Aaron Death: MLB Legend Shockingly Passes Away Weeks After Taking COVID-19 Vaccine.”

## Discussion

Our results suggest that while social media networks create and spread rumours beyond “traditional” mass media audiences in real time, general public concern is still heavily related to “traditional” media coverage. While social networks spread news and rumours quickly and widely, “traditional” journalists are still largely responsible for breaking news stories significant enough to attract broad social attention. Moreover, “traditional” media generally succeed at fact checking and preventing the spread of unfounded rumours, with hyperbolic and alarmist coverage the exception rather than the rule. However, alarmist and hyperbolic exceptions clearly spread more widely on social media than “reasonable” coverage.

### Traditional Media Coverage Is Crucial in Determining Population Level Awareness of Alleged Adverse Events

Our analysis indicates that any significant jump in google search volume (and therefore rumours of adverse events) is associated with coverage by traditional “broadcast” media channels employing professional journalists and editors. Every case study with a significant increase in Google RSV resulted from a news story that initially aired, or was covered extensively, on traditional channels.

The sterilisation case provides the exception—it developed out of online “theory crafting” by vaccine sceptical networks tying two non-associated events together in online media. This case had by far the lowest degree of correlation between Google RSV and Number of Facebook Posts (Pearson’s r = 0.22). The sterilisation claim has never been extensively explored by the traditional media as a “story” and it has not registered a large increase in Google search volume as a result. Conversely, when “traditional” media stories covered decreased fertility as a potential adverse effect of the vaccine—mostly when debunking these rumours—there was a small increase in Google search volume.

Equally, the period in which the “theory” about sterilisation was first crafted (early December) showed a significant increase in discussion of this rumour on Facebook; these discussions persisted. Possibly, rumours persist because they have not been adequately debunked in traditional media channels as they were with Hank Aaron’s death, Bell’s palsy, and syncope. The subsequent persistence of the fertility rumour on social media (and not other media) may have informed relatively high vaccine hesitancy among young women [28].

### Fact Checking and Gatekeeping Against “Fake News” Is Increasingly Important

Our analysis suggests that “traditional” journalists generally do an excellent job of promoting verified stories (such as those about clotting) and of fact checking: suppressing and debunking clearly illegitimate claims and stories. Both the Hank Aaron and Bell’s palsy rumours were treated with scepticism by the vast majority of traditional media outlets, many of whom refrained from commenting until official reports were released.

Our analysis also suggests that media reports and stories are treated as credibility markers by social media users. Vaccine-sceptical communities still rely heavily on traditional media reports to spread stories and add legitimacy to their perspective, as witnessed in the use of traditional media reports in Hank Aaron memes. The role of blogs and digital news channels also appears important, with “news reports” from non-credible sources often being shared. However, social media users appear to be aware that sharing a traditional news source is powerful, even if framed in an ironic way. This suggests that traditional journalists play an important role in framing discussions about adverse events. For the most part, they are discharging this responsibility effectively—outlining the real possibilities of adverse events and fact checking or rebuking rumours where appropriate.

Nevertheless, our study illustrates how important journalistic framing can be. The Daily Mail (UK) twice published stories with headlines associating the vaccine with Bell’s palsy or fertility problems; each of these headlines was widely shared and corresponded with a spike in Relative Search Volume on Google. Similarly, a “newsworthy” but misleading headline “Hank Aaron Death: MLB Legend Shockingly Passes Away Weeks After Taking COVID-19 Vaccine” from Republicworld.com was shared internationally across vaccine-sceptical networks, attaing far greater reach than more accurate stories. This finding resonates with recent research that suggests inaccurate reports spread more rapidly than accurate reports on social media [29–31].

### Information Spreads Through Interest Networks Unconstrained by National Boundaries

Finally, the way in which information disseminates is now largely unfettered by national points of origin and has more to do with specific networks, communities, and language groups. For both “sterilization” and “Hank Aaron’s death” vaccine-sceptical communities utilised international sources to build evidence for adverse events—which they then redistributed among their international networks. The rumours about Bell’s palsy and fainting originated from local news reports. However, they primarily spread through social networks organised around shared interests (such as natural health) extending across countries. The discussion around fainting is particularly instructive: the initial story was not extensively covered on international news, but spread globally via social media.

While this study endorses Bhattacharyya et al.’s [4] point that global media is more important than local media in disseminating rumours about adverse events, it also suggests that we can’t exclude local media from becoming a global source. Moreover, contrary to the assumption that “the intensity of the information decays as it propagates through the network from one node to its neighbouring nodes, much like ripples in a pond,” [4] social media networks can actively merge stories to create a “superposition” of two ripples; distributing that perspective in new directions with continued momentum. Consequently, information may travel without decay and can even be amplified if not contradicted by a more credible or authoritative source.

### Limitations

This study has a number of limitations. Our ethical conditions and CrowdTangle’s limitations precluded tracking private activity on Facebook. This skews our results toward posts that people are prepared to share publicly, which could explain the prevalence of traditional news stories and sources. Private social media discussions may have prompted activity around further rumoured adverse events not covered here. However, if this did occur (as seems to have happened in the “sterilisation” case study), activities were generally not significant enough to produce a notable increase in Relative Search Volume for that topic on Google search [32].

The language parsing in our search tools also imposed a limitation. While searching topics on Google Trends includes “terms that share the same concept in any language” [33], Google provides no information about how this parsing takes place or what terms it includes. As such, we rely on Google’s integrity, ability, and accuracy. Also, CrowdTangle and Factiva are limited by the terms used in the search (and therefore by the language used); our social media data functionally excludes many non-English results. So while our collection of rumours was broadly global, our investigation of spread was limited to the corpus of English language public Facebook posts and newspapers. Future work could explore rumour spread in more specific languages.

The “black swan” nature of the COVID-19 pandemic also limits the replicability of our method and findings. Google Trends worked because COVID-19 vaccines were a topic of relatively unique global importance and prominence, transcending national boundaries. Similarly, the release of the first batch of vaccines in front of a captivated global audience represented a “period of [public] mobilization” during which the media and the public are more careful than usual about interrogating claims to truth around crucial topics [34]. Traditional media may not always be so rigorous with less prominent or impactful topics.

Google and CrowdTangle make it difficult to guarantee replicability because their data sorting and ranking mechanisms may have undisclosed biases or change without notice. The ongoing removal of various social media posts, blogs and channels by platforms or individuals means that some posts can disappear. We cannot say our data is exhaustive or definitive of global information exchange—we are looking at public facing information people are prepared to share in one particular (but widespread) language. Nevertheless, our method has uncovered generally relevant rumours and faithfully identified their likely point of origin.

### Conclusion

Studies of social media, information seeking, and news sharing can provide valuable insights for understanding and addressing misinformation about COVID-19 vaccination. Our case studies demonstrate that traditional media continue to play an important role as authoritative sources. While committed anti-vaccination activists can circumvent traditional media and successfully generate spikes in interest and coverage, even these populations look to traditional media sources as markers of credibility. Egregious reportage and sub-editing by publications clearly encourages ongoing circulation of misinformation—and the Internet has facilitated the proliferation of less rigorous and credible reporting.

It is equally concerning that hyperbolic reporting tends to be shared more widely, so writers and platforms incentivised by clicks may exaggerate claims. Ideally, journalists and editors should be counter-incentivised to report the news as accurately as possible. Employing markers of source credibility on social media would provide a valuable countermeasure; platforms could deploy weighted peer evaluations on stories and posts, displaying the comments and sentiments of people with demonstrated expertise most prominently [35].

Traditional journalism (still) plays a crucial role in fact checking public health discussions. Ideally journalists would monitor social media for rumours about adverse events and engage relevant scientists to examine their veracity before publishing a story. However, scientists and health professionals should (also) look to promote their own perspectives when they believe that a story about adverse events needs clarifying. Our study suggests that such activities have a significant impact on the spread of rumours about adverse events, and therefore points to a need for available experts to help correct misinformation.
